# An Integrated Intrusion Detection Model of Cluster-Based Wireless Sensor Network

**DOI:** 10.1371/journal.pone.0139513

**Published:** 2015-10-08

**Authors:** Xuemei Sun, Bo Yan, Xinzhong Zhang, Chuitian Rong

**Affiliations:** School of Computer Science and Software, Tianjin Polytechnic University, Tianjin, 300387,China; Nankai University, CHINA

## Abstract

Considering wireless sensor network characteristics, this paper combines anomaly and mis-use detection and proposes an integrated detection model of cluster-based wireless sensor network, aiming at enhancing detection rate and reducing false rate. Adaboost algorithm with hierarchical structures is used for anomaly detection of sensor nodes, cluster-head nodes and Sink nodes. Cultural-Algorithm and Artificial-Fish–Swarm-Algorithm optimized Back Propagation is applied to mis-use detection of Sink node. Plenty of simulation demonstrates that this integrated model has a strong performance of intrusion detection.

## Introduction

Compared with traditional network, wireless sensor network [1] (WSN) has a more open disposal area and more vulnerable wireless communication channel, accordingly it is more susceptible to network attack and hijack. Therefore, the study on security is evidently important. At present, the academic has put more emphasis on encryption, security protocol, identity authentication, security routing and other preventive systems for WSN security. Undoubtedly, these are the fundamentals of WSN secure communication, but only for the passive defense use. For example, tiny encryption algorithm (TEA)-based data encrypt protocol [2], integrated advanced encryption standard (AES) algorithm, RSA public key system and digital autograph secure Ad hoc on-demand distance vector (SAODV) security routing protocol [3]. Along with the development of network technology, network attack is more sophisticated than ever. Above-mentioned passive defense systems, due to their real time deficiency, are always incapable of confronting new attacks. Intrusion detection technology, as an active defense, on the contrary, can be very quick on discovering and reporting anomaly and attack incidents. It demonstrates more responsiveness and adaptability, and consequently, can be applied as the supplement and a second line of defense for passive prevention security systems.

Intrusion detection can be categorized with anomaly and mis-use detection. Anomaly detection is a process of finding out abnormal ones from a great deal of data running through system, based on users’ common operations, and reporting to administrators. Mis-use detection is primarily used to recognize any specific attack.

At present, achievements of WSN intrusion detection study are limited home and abroad. Reference [4] brings forward a novel WSN intrusion detection framework, which is light weight and self-learning and can identify unknown attacks. Reference [5] proposes a detection system based on Adaboost with hierarchical structures. Through increasing weight variation and searching optimal classifier, it enhances the accuracy and responsiveness. However, it doesn’t perform dimension reduction and the training of classifier is prolonged. Reference [6] comes up with an integrated intrusion detection system for cluster-based WSN. It gives different solutions based on ordinary nodes, cluster head nodes and sink nodes, with a reference on different types of WSN and their resources. Combining anomaly and mis-use detection, it increases detection rate and accuracy. The defect is that anomaly detection algorithm is over-simplified. Reference [7] gives a detection system based on artificial immune genetic algorithm back propagation, but it lacks of cluster-based WSN and no division of nodes accordingly. Reference [8] proposes an online anomaly detection of WSN relying on scalable hyper-grid k-NN (k-nearest-neighbor) algorithm. Reference [9] analyzes the problem of intrusion detection in a Gaussian-distributed WSN by characterizing the detection probability with respect to the application requirements and the network parameters under both single-sensing detection and multiple-sensing detection scenarios.

Taking weak performance and power of sensor into consideration, this paper proposes an integrated intrusion detection model of cluster-based WSN. It combines the advantages of both anomaly and mis-use detection to enhance detection rate and reduce false rate. The proposed model includes both anomaly detection of sensor nodes, cluster head nodes and Sink nodes, and mis-use detection of only Sink nodes. Intrusion data from original network has too many dimensions and a large volume. If all features are used in classification, a great number of useless features not only weaken the performance of detection system but also raise power consumption of nodes. The paper applies PCA (principal component analysis) to dimension reduction, so as to bring down data storage volume and network power consumption. Adaboost algorithm is used in anomaly detection to construct two-pass classifier. Hierarchical structures help to reduce power consumption further, which alleviates the deficiency that sensor is always weak in performance and power. Cultural algorithm based on evolutionary algorithm of the principle of human society is composed of population space and belief space. And it improves the evolutionary efficiency by using the double evolutionary mechanism which is applied to the optimization of the problem[10]. So, the paper applies Cultural Algorithm and Artificial-Fish-Swarm Algorithm optimized Back Propagation (CA-AFSA-BP) to mis-use detection.

## Intrusion Detection Model of WSN

**Fig 1:**
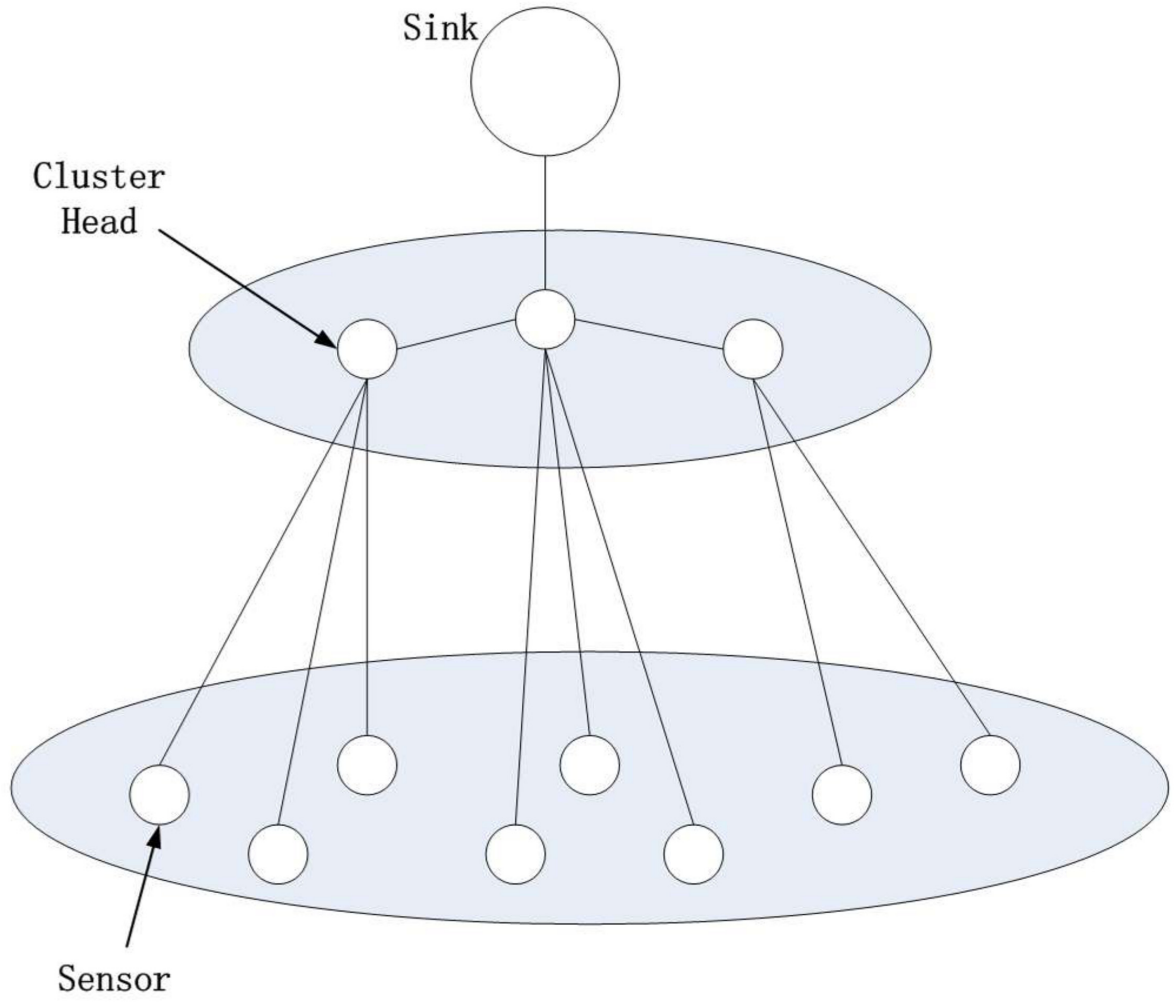
Cluster-based WSN.

This paper proposes an integrated intrusion detection model for cluster-based WSN. AS shown in Fig 1, each cluster consists of one cluster head (CH) and several sensor nodes (SN). Inside each cluster, CH takes charge of communications with ordinary nodes and Sink nodes. A large number of communications between sensor nodes are restricted inside each cluster and long distance communication is cut down. Therefore, routing is less complicated and easier to manage, and efficiency is higher. Network topology of this kind is applied to WSN in the paper.

**Fig 2:**
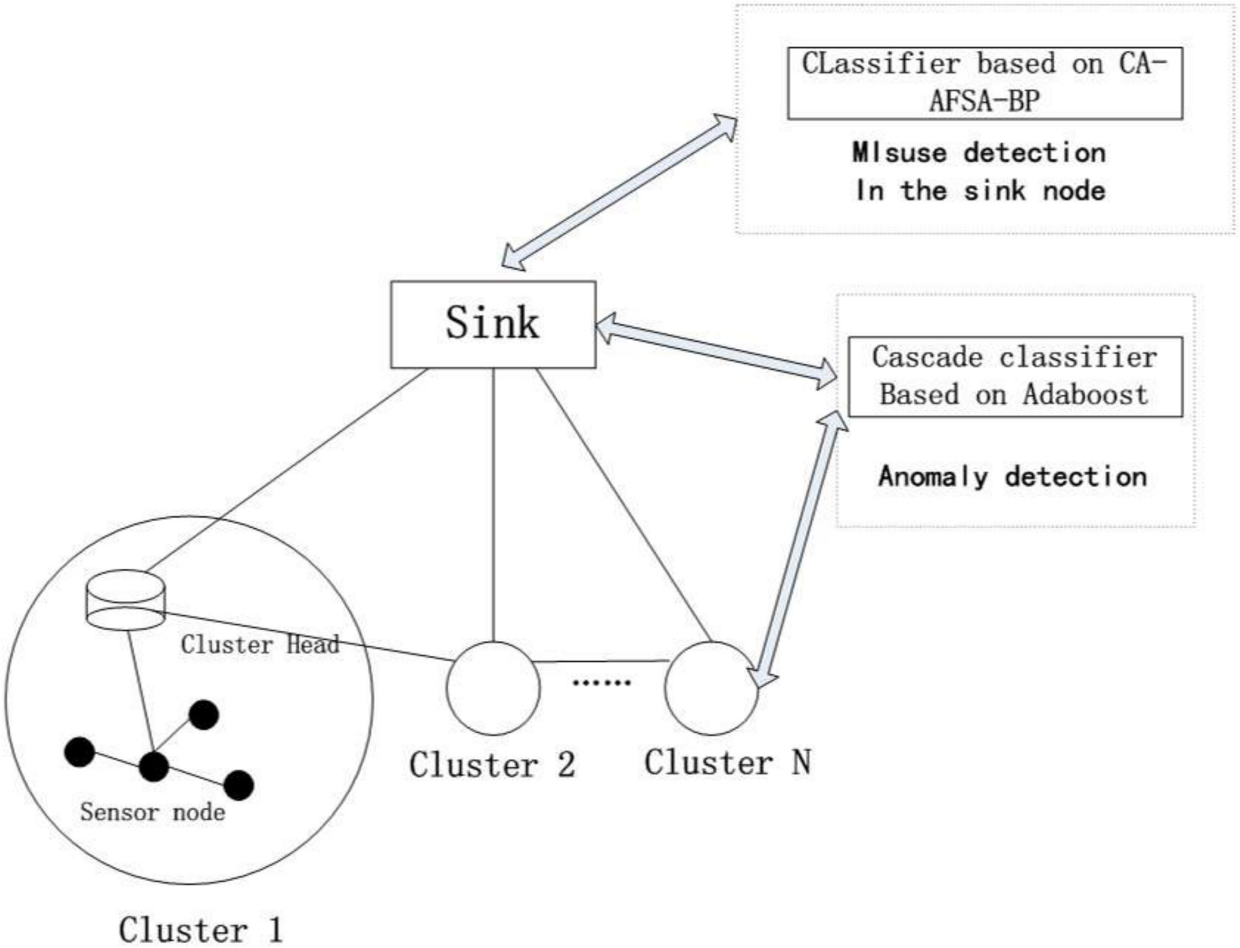
The integrated intrusion detection model of cluster-based WSN.

The integrated intrusion detection is demonstrated in Fig 2. Adaboost with a hierarchical structure is adopted for anomaly detection of sensor nodes, cluster head nodes and Sink nodes, while Cultural Algorithm, Artificial Fish Swarm Algorithm, Back Propagation is applied for mis-use detection of only Sink nodes.

### 2.1 Anomaly Detection System inside Clusters

Due to limited computing performance of WSN, if strong classifier is applied on sensor nodes, a lot of time and energy will be spent. At the same time, intrusion detection requires responsiveness. To confront this challenge, Adaboost with hierarchical structures[5] is suggested for anomaly detection of cluster-based WSN. Many weak classifiers are integrated into a strong one that is highly stable and adaptable. Hierarchical structures help to screen out most negative samples easily at first levels and therefore enhance detection rate.

**Fig 3:**
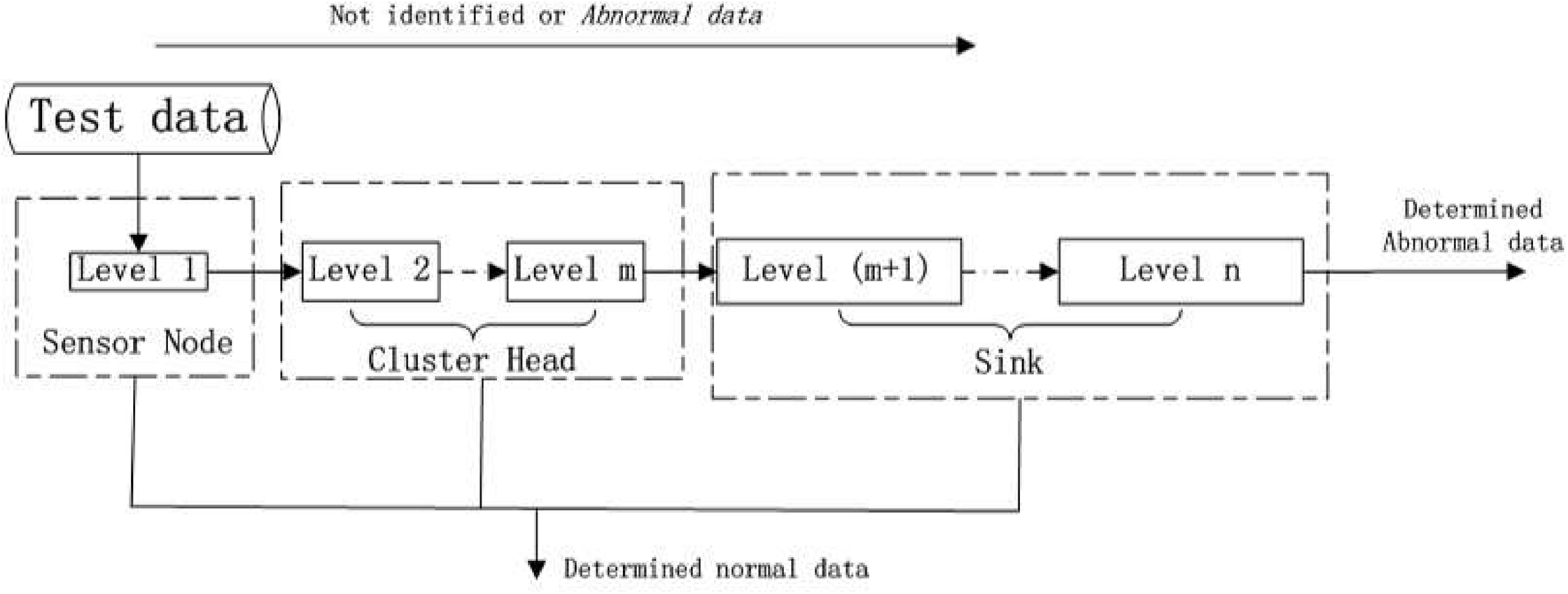
Cluster-based anomaly detection flow.

Fig 3 portrays an anomaly detection flow. Well trained Adaboost with hierarchical structures is applied to sensor nodes, cluster head nodes and Sink nodes. If data is determined as normal ones at each level of classifier, it remains to be normal data for all next levels. If it is defined as anomaly or uncertainty, detection shall be performed once again at following levels. First level classifier is deployed at sensor nodes. Due to computing limit of sensor nodes, classifier at this level has simpler structure and fewer features. With initial parameters given, high detection rate can be achieved, but at the same time errors rise and some normal data can be misjudged as anomaly. Secondary and the mth level classifiers work at cluster head nodes. Stronger computing capacity allows the use of more features and more complicated structures, so anomaly similar to normal data can be detected. The outcome of classification at cluster head nodes is passed to Sink nodes. The m+1th to nth level classifiers function at Sink nodes, which perform detection once more of anomaly and uncertainty. If data is determined as anomaly, alarm will be made. If data is defined as uncertainty, it will return to the very beginning classifier for a second round detection, until final outcome is obtained.

During the process of real time intrusion detection, anomaly accounts for a quite small percentage of all data. Most of normal data is ruled out for front levels of detection, with only few anomaly data to be left through all levels. Calculation is greatly reduced to satisfy the needs of WSN.

### 2.2 Mis-Use Detection of Sink Nodes

**Fig 4:**
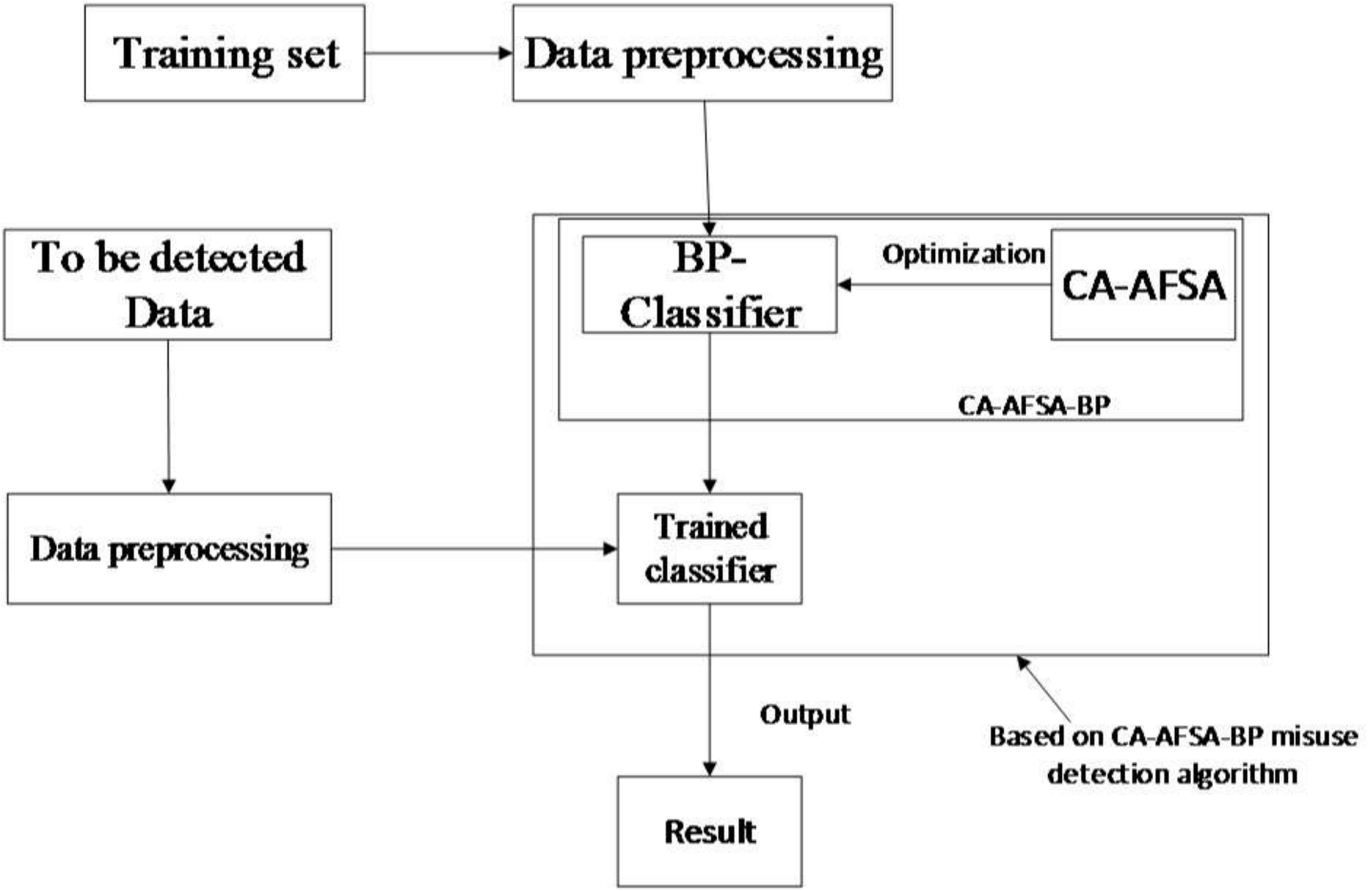
CA-AFSA-BP mis-use detection of flow.

The CA-AFSA-BP algorithm is applied to mis-use detection of Sink nodes. Sink nodes receive data from cluster heads or from outside. Optimized classifier is suggested to identify specific attacks and response accordingly. As shown in Fig 4, training is made through CA-AFSA-optimized BP after pretreatment of data. Then, well-trained mis-use classifier is able to distinguish normal network data from intrusion incidents. It can further identify the specific intrusion type.

## Method of Intrusion Detection Algorithms of WSN

### 3.1 Dimension Reduction of Network Intrusion Data by PCA

Network intrusion data has high dimensions, whose number is generally over 40. On the one hand, most of network data is irrelevant of intrusion; on the other hand, WSN is limited in resources. In other words, much nodes calculation is wasted, and the accuracy and efficiency of detection system falls. PCA[11] gives analysis on high-dimensional data and then helps to decrease its dimensions. This paper also applies PCA to dimension reduction through picking up the most relevant and crucial features from detection data. Main process is as follows:

Step 1: standardize original data

Step 2: calculate covariance matrix

Step 3: obtain feature vector and value through analysis of covariance matrix

Step 4: determine contribution rate according to Feature value, choose vectors whose accumulated variance contribution rate is over 95% as principal component, and obtain PCA matrix from chosen vectors.

### 3.2 Anomaly Detection inside Clusters by Adaboost

Adaboost[12–13] is an important algorithm of machine learning and is widely considered as one of ten classic algorithms in data mining. It is iterative. The main concept is to train various weak classifiers for the same training set and combine them as a stronger classifier. Adaboost puts weight to every trained sample. The weight represents the probability a certain weak classifier selects it. If a sample is correctly classified by this classifier, its weight will fall. The selection probability of it into next weak classifiers is also smaller. Otherwise, its weight will rise and the selection probability of it to next round training is larger. In a word, Adaboost is able to concentrate on those samples that can be easily mis-classified. But the use of Adaboost in intrusion detection is confronted with high dimensions of data. In order to avoid much time waste on useless features in training, the paper combines PCA and Adaboost, and forms a strong classifier to improve anomaly detection rate and reduce false rate.

Adaboost can be described as follows:

Input: Choose training set S = {(x_1_, y_1_),……,(x_m_, y_m_)}, where x_i_∈X, y_i_∈Y = {-1,1}, 1 is normal sample and -1 is anomaly, i = 1,2,3,……,m;

Maximum iteration times T is the number of weak classifiers

Output: A strong classifier made from T weak classifiers

Begin

    Initialize the weight of sample x_i_ $\left\{ \begin{array}{l} \text{nomal sample}\;w_{t,i}=1/2p\\ \text{anomaly sample}\;w_{t,i}=1/2q \end{array}\right.$, where p is the number of normal samples and q is the number of anomaly samples.

While end is not satisfied Do

Begin

    For 1 to T, // train classifier *h*_*t*_ by T times iteration and calculate training error,

      h_t_(x_i_)∈{-1,1}, $\varepsilon_{t}=\sum_{i}w_{t,i}I({\text{y}}_{i}\neq {\text{h}}_{i}({\text{x}}_{i}))$

      if(*ε*_*t*_ = 0||*ε*_*t*_ ≥0.5)

        break;

      then

        refresh *w*_*t*,*I*_, and perform next training $w_{t+}{}_{1,i}=\frac{w_{t,i}}{Z_{t}}\cdot e^{-a_{t}y_{i}h_{i}({\text{x}}_{i})}$, where *Z*_*t*_ is normalized coefficient, $a_{t}=\frac{1}{2}\text{log}\frac{1-\varepsilon_{t}}{\varepsilon_{t}}$。

    End For

End

    Output strong classifier end function $H(\text{x})=\text{sgn}(\sum_{t=1}^{T}a_{t}\;\text{h}(\text{x})-\theta )$, where sgn is sign function; Classify samples according to the value of H(x). *θ* is end threshold, and the initial value is the average of all weak classifiers $\theta =\frac{1}{T}\sum_{t=1}^{T}a_{t}$。

End

### 3.3 Mis-use Detection of Sink Nodes by CA-AFSA-BP

**Fig 5:**
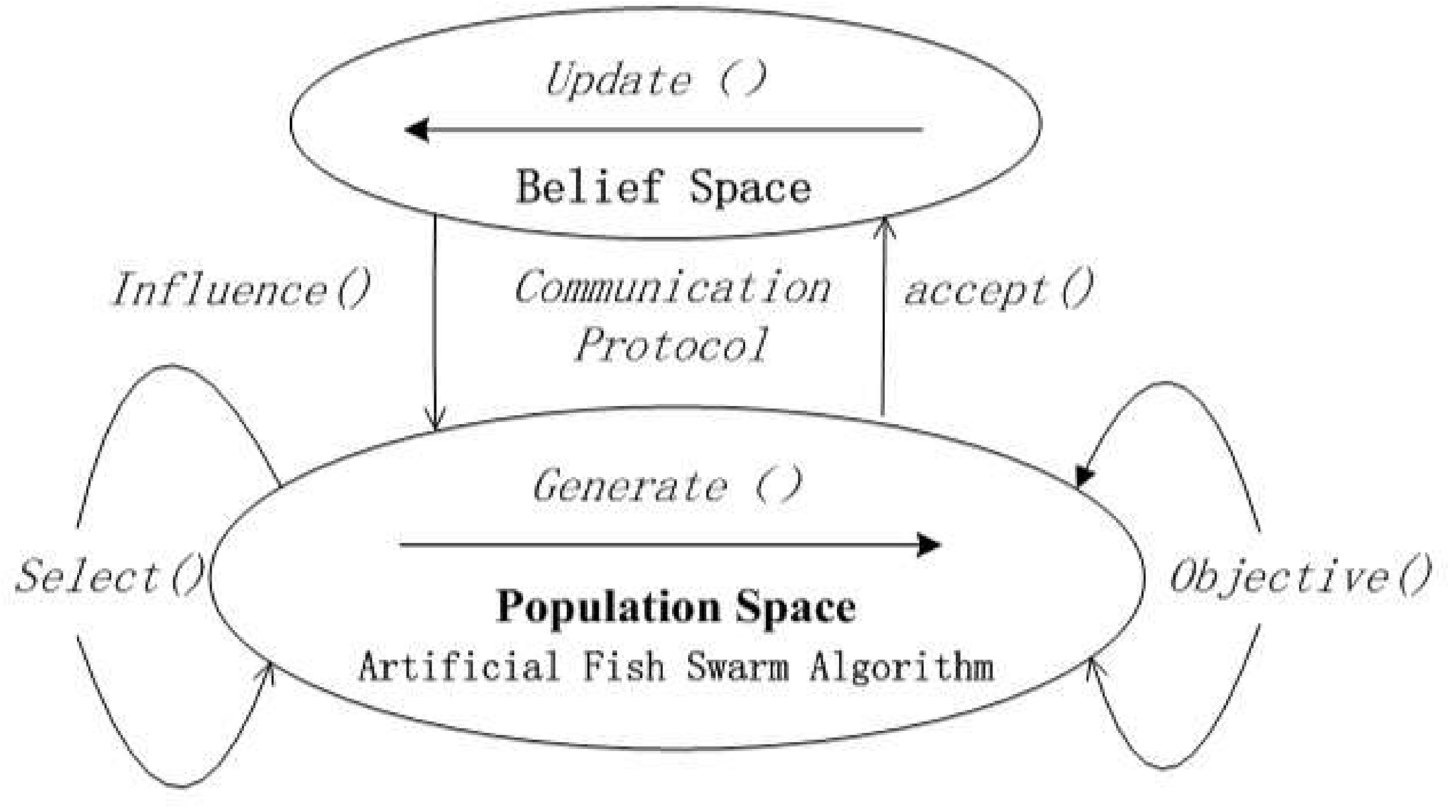
A global framework of CA-AFSA-BP.

The paper trains BP neural network through CA and AFSA. CA[14]is a dual evolutionary system consisting of population space and belief space. It contains three major elements: population space, belief space and communication protocol. Population space, from a microscopic angle, simulates evolution process of individual living things abiding by a certain code of conduct. Belief space, from a macroscopic angle, simulates evolution process, such as the form, inheritance, and comparison of cultures. Population space and belief space, on the one hand, are independent of each other, and on the other hand, are interdependent and mutually promotive. Two spaces exchange messages through communication protocol. Effective selection and management of data will help to guide the evolutionary process of population space. The basic idea of CA-AFSA-BP is to take advantage of dual evolutionary strategy of CA and integrate AFSA as one evolution step of population space into CA. Fig 5 shows a global framework. In this way, CA-AFSA-BP is integrated.

#### 3.3.1 BP Neural Network

**Fig 6:**
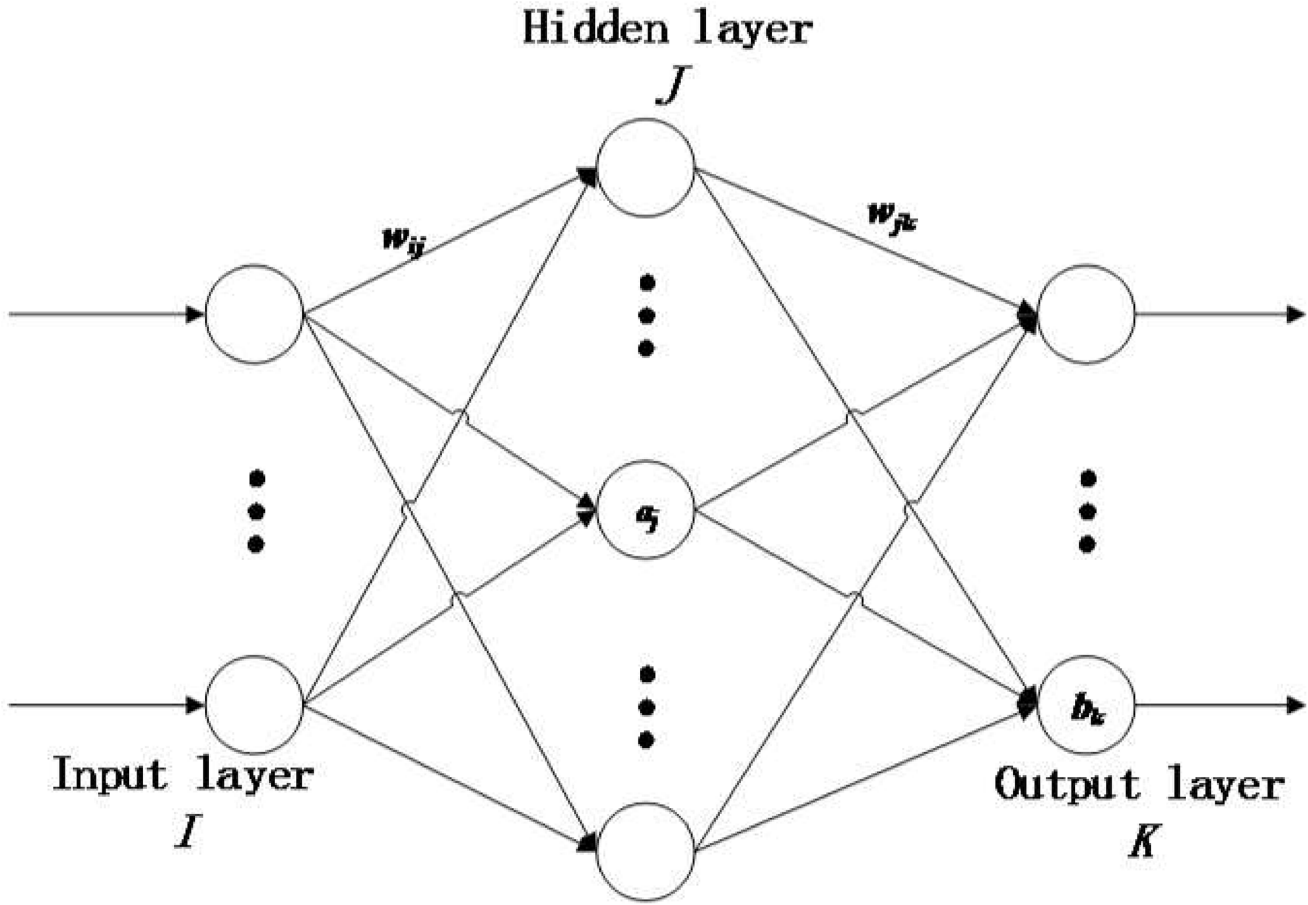
BP neural network.

BP is a multi-layer feed-forward neural network and applies algorithms of backward propagation of error. As shown in Fig 6, the network consists of three layers: input I, hide J and output K. By the activation of input samples, BP obtains the gradient of the weight so as to make output become closer to the desired.

BP Neural Network can be illustrated as follows:

Step 1: Initialize network and learning parameters, determine transfer function of neurons and network structure (i.e. network layers and number of nodes on each layer), and assign proper weight.

Initialize network: From list entries (*X*, *Y*), determine numbers of nodes on input layer *n*, on hide layer *l*, and on output layer *m*, initialize connection weights between neurons of input, hide and output: *w*_*ij*_, *w*_*jk*_, where 1 ≤ *i* ≤ *n*, 1 ≤ *j* ≤ *l*, 1 ≤ *k* ≤ *m*, initialize threshold of hide layer *a*,output layer *b*, assign learning rate and active function of neurons.

Step 2: Calculate hide layer output From input vector *X*, connection weight *w*_*ij*_ between input layer and hide layer, threshold of hide *α*, calculate output *H* of hide layer.

$$Yo_{j}=\sum_{i=1}^{n}w_{ij}x_{i}-a_{j}\;j=1,2,\ldots ,l$$ (1)

$$H_{j}=f(Yo_{j})\;j=1,2,\ldots ,l$$ (2)

In the equation, *l* is the number of nodes on hide layer, *f* is the active function of hide layer.

Step 3: Calculate output layer output. From output *H* of hide layer, connection weight *w*_*jk*_, and threshold *b*, compute predictive output of BP neural network *O*.

$$Yi_{k}=\sum_{j-1}^{l}H_{j}w_{jk}-b_{k}\;k=1,2,\ldots ,m$$ (3)

$$O_{k}=f(y_{k})\;k=1,2,\ldots ,m$$ (4)

In the equation, *k* is the number of neurons in output layer.

Step 4: Calculate error. From network predictive output *O* and expected output *Y*, compute network predictive error *e*.

$$e=\frac{1}{2}\sum_{k=1}^{m}{\left(Y_{k}-O_{k}\right)}^{2}$$ (5)

Step 5: Compute partial derivatives *δ*_*k*_ and *δ*_*j*_ of error functions with respect to neurons of output layer.

Step 6: Modify connecting weight *w*_*jk*._, from *δ*_*k*_ of neurons of output layer and neurons of hide layer,

$$\Delta w_{jk}=-\mu \frac{\partial e}{\partial w_{jk}}=\mu \delta_{k}H_{j}$$ (6)

$$w_{jk}^{N+1}=w_{jk}^{N}+\eta \delta_{k}H_{j}$$ (7)

Step 7: Modify connecting weigh, from *δ*_*j*_ of neurons of hide layer and neurons of input layer, t.

$$\Delta w_{ij}=-\mu \frac{\partial e}{\partial w_{ij}}=-\mu \frac{\partial e}{\partial Yo_{j}}\frac{\partial Yo_{j}}{\partial w_{ij}}=\delta_{h}x_{i}$$ (8)

$$w_{ij}^{N+1}=w_{ij}^{N}+\eta \delta_{j}x_{i}$$ (9)

Step 8: Compute global error.

$$E=\frac{1}{2Num}\sum_{num=1}^{Num}\sum_{k=1}^{m}{\left(Y_{k}-O_{k}\right)}^{2}$$ (10)

where *Num* is the number of samples.

Step 9: Determine if network error meets the requirements. When error is smaller than the designed accuracy, or times of learning reach the maximum, the computation ends. Otherwise, choose next learning sample and a corresponding desired output, and go back to step 2 into the next round of learning.

#### 3.3.2 Evolutionary Strategy of Population Space

AFSA is adopted as evolutionary strategy of population space. Because of many flaws of BP, such as it tends to get local optimums, low speed of astringency, and network structure is difficult to determine, the paper optimizes BP with CA-AFSA. In population space, AFSA is applied to BP training and in belief space inheritance algorithm is used. AFSA is a bio-inspired intelligent optimizing algorithm brought up by Xiaolei, Lee et al. [15]in 2002. The basic idea is that in a body of water, more food attracts more fish to get together. By simulating fish swarm prey behavior, optimal goal can be reached. Basic behaviors of fish include prey, swarm, follow and move. It uses the fish bottom behavior process and finds the global optimal.

**Determine AF swarm parameters**: the position of AF can be expressed with vector *X*_*p*_ = (*x*_*1*_, *x*_*2*_, *… x*_*i*_, *…*, *x*_*n*_), where *x*_*i*_ (*i* = 1, 2, 3, …, *n*) is the variable to be searched for the optimal value; food consistence *Y* at present position can be represented by *Y* = *f(X)*, in which Y is objective function; *d*_*pq*_ is the distance between individual AF *X*_*p*_ and fish *X*_*q*_; *Visual* is the visual distance of AF; *step* represents the step length of AF; *δ* is the crowd factor.

The key to optimizing BP is to set up AF model. In neural network two weight matrix [*w*_*ij*_], [*w*_*jk*_]and two threshold vectors [*a*_*j*_], [*b*_*k*_] are optimal parameters. Each AF represents a neural network, therefore each optimal parameter of neural network can transform into AF, which is displayed as:

$$X=X([w_{ij}],[w_{jk}],[a_{j}],[b_{k}])$$ (11)

Food consistence at present position *Y* is:

$$Y=\frac{1}{E}$$ (12)

where *E* is global error of neural network.

The distance between individual AF *X*_*p*_ and fish *X*_*q*_ is represented as:

$$d_{pq}=\sum_{i=1}^{n}\sum_{j=1}^{l}[w_{ij}(p)-w_{ij}(q){]}^{2}+\sum_{j=1}^{l}\sum_{k=1}^{m}[w_{jk}(p)-w_{jk}(q){]}^{2}+\sum_{j=1}^{l}{\left[a(p)-a(q)\right]}^{2}+\sum_{k=1}^{m}{\left[b(p)-b(q)\right]}^{2}$$ (13)

Prey: Let *X*_*p*_ express the AF states at present and select a state *X*_*q*_ in its visual (*d*_*pq*_≤*Visual*) randomly, if food consistence at state *X*_*p*_ is smaller than that at state *X*_*q*_, i.e. *Y*_*p*_ *<Y*_*q*_, in the maximum problem, go a step forward in the direction; otherwise, select a state *X*_*q*_ and justify whether it meets the forward requirement or not, repeat *try-number* times, if it not yet meet, go a step randomly and take Eq (15).

$$w_{ij}(next)=w_{ij}(p)+rand\cdot step\cdot \frac{w_{ij}(q)-w_{ij}(p)}{d_{pq}}\;({\text{Y}}_{q}>Y_{p})$$ (14)

$$w_{ij}(next)=w_{ij}(p)+rand\cdot step\;({\text{Y}}_{q}<Y_{p})$$ (15)

*w*_*jk*_, *a*, *b* are likewise; rand∈[0,1] is a random number.

Swarm: Let *X*_*p*_ express the AF states at present, and search in its visual (*d*_*pq*_≤*Visual*) the number of its fellows *N*_*f*_ and the center position *X*_*center*_ with the food consistence at center position *Y*_*center*_. If *Y*_*center*_/*N*_*f*_ >*δY*_*p*_, which means that food at center position are plenty and fellows are not crowd, forward a step to the fellow center according to Eq (17); otherwise, execute the pray behavior.

$$w_{ij}(center)=\sum_{num=0}^{N_{f}}w_{ij}\;/\;N_{f}$$ (16)

$$w_{ij}(next)=\left\{ \begin{array}{ll} w_{ij}(p)+rand\cdot step\cdot \frac{w_{ij}(center)-w_{ij}(p)}{d_{center,p}} & \;({\text{Y}}_{center}\;/\;N_{f}>\delta Y_{i})\\ prey & \;({\text{Y}}_{center}\;/\;N_{f}\leq \delta Y_{i}) \end{array}\right.$$ (17)

*w*_*jk*_, *a*, *b* are likewise.

Follow: Let *X*_*p*_ express the AF states at present, and search in its visual (*d*_*pq*_≤*Visual*) the number of its fellows *N*_*f*_ and the state *X*_*max*_ which has the largest food consistence. If *Y*_*max*_/*N*_*f*_ *>δY*_*p*_, which means that the fellow has high food consistence and the surrounding is not very crowd, go a step forward to the fellow, and perform Equation (18); otherwise, execute the pray behavior.

$$w_{ij}(next)=\left\{ \begin{array}{ll} w_{ij}(p)+rand\cdot step\cdot \frac{w_{ij}(\text{max})-w_{ij}(p)}{d_{\text{max},p}} & \;({\text{Y}}_{\text{max}}\;/\;N_{f}>\delta Y_{i})\\ prey & \;({\text{Y}}_{\text{max}}\;/\;N_{f}\leq \delta Y_{i}) \end{array}\right.$$

*w*_*jk*_, *a*, *b* are likewise.

Bulletin: Bulletin is used to record optimal individual AF state. After each AF finishes its search, the food consistence it finds will be compared with the optimal on the bulletin. If the food consistence that one AF finds is bigger, its state will take the top place on the bulletin and therefore AF is sorted.

#### 3.3.3 Evolutionary Strategy of Belief Space

Belief space is the other evolutionary process, independent of population space. The paper introduces genetics algorithm as the evolutionary strategy of belief space, which forms dual evolutionary system, together with that of population space. Elite set stored in belief space continuously evolves by generations with genetic operators of selection, crossover and mutation. Exchanges with population space through communication protocol help to make two spaces interdependent and mutually promotive.

Initialize belief space. Take the same code for belief space as those for population space. Let population in belief space 30% of that in population space. Assign front 30% population on bulletin with belief space and note as *pop_size_belief*.

Design genetic operators. Selection: select present population fitness according to individual fitness that has the optimal food consistence in population space (code as *fit*_*max*_). Keep the individual fitness and copy into next generation. Select a number of individuals by roulette wheel and perform crossover and mutation. Take them into generations. Note fitness of individual *i* as *fit*_*i*_. The probability of *p*_*i*_ that *i* can be selected in roulette wheel is: select individuals whose number is 2 (*pop_size_belief* -1) by roulette wheel. Operate with crossover and mutation and sort by fitness. Make sure population size stays constant and select a proper number of optimal individuals into next generations. Crossover: introduce single point crossover. Replace and reform partial structures of two individuals of parent generations to create new individual. Execute crossover by assigned probability *p*_*c*._ Mutation: create new next generation from crossover. Determine whether to perform mutation with assigned probability *p*_*m*._

#### 3.3.4 Communication Protocol

Communication protocol is a channel of interchange between two spaces of Cultural Algorithm. Constant communication of two spaces is made through *accept* and *influence*. Updated search results of each space are timely fed back and exchanged, so as to share information.

Accept. Update on a regular basis is adopted during evolution of population space. When *AcceptStep* runs by Artificial Fish Swarm algorithm, worst individual in belief space will be replaced by optimal individual in global population space at present

Influence. This paper chooses dynamic influence occasions, which makes the influence of belief space on population space increase with growing evolution generations. Assign a dynamic variable *InfluenceStep*. When *InfluenceStep* runs among population in belief space, a number of individuals with relatively worse fitness in population space will be replaced by same number of individuals with better fitness in belief space. The value of *InfluenceStep* can be obtained by following equation:*InflunceStep = N1+N2*(*iter*_*max*_ − *CurrentStep) / iter*_*max*._ Where *N1*, *N2* is constant numbers, *iter*_*max*_ is the maximum evolution generation, and *CurrentStep* is the current evolution generation of population space. Let *N1*, *N2* be 2 and 5 respectively.

#### 3.3.5 CA-AFSA-BP Program

CA-AFSA-BP program is as follows:

Input: initialize the value and threshold of BP network, the size of population space *pop size*, maximum iteration times *iter*_*max*_, visual distance of individual AF *Visual*, the maximal step length of AF *step*, the crowd factor *δ* and other parameters.

Output: BP neural network that meets requirements

Begin

    CurrentStep←0; / / 0 generation

    Initialize population space and belief space;

While not satisfy end term Do

Begin

    For 1 to *pop_size* Do // execute AFSA in population space

      Execute prey;

      Execute Swarm;

      Execute follow;

      Calculate the food consistence of each individual AF at present state, i.e. current fitness,

      Refresh bulletin;

    End For

    If CurrentStep % AcceptStep = 0 then

      Execute accept();

    Execute selection, and select (*pop_size_belief* -1) individual set;

    For 1 to *pop_size_belief* Do

      Randomly create operation probability *p*_*temp*_ = *rand*;

      If (*p*_*temp*_≤*p*_*c*_) Then

        Meet crossover probability term. Execute crossover to set *i*;

      If(*p*_*temp*_≤*p*_*m*_) Then

        Meet mutation probability term. Execute mutation to new next generation;

    End For

    Keep top (*pop_size_belief* -1) individuals with best fitness to next generation;

    If *CurrentStep % InflunceSetp* = 0 Then

      Execute *influence*();

    *CurrentStep*←*CurrentStep* + 1;

End

    Output optimal neural network;

End

## Results and Discussion

### 4.1 Data Selection

In this paper, training and test data set is KDD CUP 99, packed up by Columbia University from MIT Lincoln’s collection of simulated military network environment data of American Defense Ministry. The data set contains 38 types of intrusions, which can be categorized into 4 different attacks: DoS, R2L, U2R and Probe, and also normal incidents. Each line of data set has feature value of 41 dimensions and type value of 1 dimension.

This paper takes 10% KDD CUP 99 data set and selects 5000 data as training samples of anomaly and mis-use detection and 1000 data as test samples, as shown in Table 1. Data pretreatment is also taken for anomaly and mis-use detection.

**Table 1:** Selection of training and test samples.

	Normal	Probe	DoS	U2R	R2L	Total
Anomaly detection training set	3000	2000				5000
Anomaly detection test set	2000	1000				3000
Mis-use detection training set	805	767	3133	52	243	5000
Mis-use detection test set	483	460	1880	31	146	3000

### 4.2 Data Features Extraction

Experiment of this paper uses MATLAB simulation software and employs PCA for features dimension reduction of training samples of anomaly and mis-use detection. Same PCA matrix is used for dimension reduction in test samples. Experiment shows that when threshold is 97%, 18 from 41 dimensions of anomaly detection and 14 of mis-use detection are selected, best results can be achieved.

### 4.3 Intrusion Detection Simulation

#### 4.3.1 Evaluation Index

There are three performance indexes to evaluate intrusion detection

Detection rate (DR) which can be calculated as in equation:

$$\text{Detection rate}=\frac{\text{Number of detected attacks}}{\text{Number of attacks}}\times 100\%$$

False positive rate which can be calculated as in equation:

$$\text{False positive rate}=\frac{\text{Number of misclassified connections}}{\text{Number of normal connections}}\times 100\%$$

Accuracy which can be calculated as in equation:

$$\text{Accuracy}=\frac{\text{Number of correct classified connections}}{\text{Number of connections}}\times 100\%$$

#### 4.3.2 Algorithm Simulation

In the paper, two experiments of algorithm simulation are conducted. Experiment 1 is taken to examine the performance of cluster-based anomaly detection and experiment 2 is to observe the performance of mis-use detection of nodes.

#### Experiment 1

Adaboost with four-hierarchical structure for anomaly detection is adopted in the paper. First, use training data to train cluster-based classifiers, and then exploit test data to examine classifiers performance. Results are shown in Table 2. When applying Adaboost with hierarchical structures for cluster-based anomaly detection, on the first level, 30.3%, i.e. 606 normal data are misreported as intrusion and 1394 normal data are ruled out. In this way, the next levels are exempted from interfere of many normal data. After several levels of screening and the increase of features, false rate declines to less than 2% in the end. Results prove that the algorithm is feasible in wireless sensor network.

**Table 2:** Cluster-based anomaly detection results.

Level	Position	The number of features	*DR*(%)	*FP*(%)
Level 1	Sensor node	5	100	30.30
Level 2	Cluster head	10	96.62	29.45
Level 3	Sink node	18	92.47	8.56
Level 4	Sink node	18	92.40	1.66

Detection rate (DR) and False Rate (FR) of Adaboost anomaly detection with and without hierarchical structures are compared as Table 3. It shows without hierarchical structures, FR in anomaly detection of WSN is much higher at 8.5%. Therefore, Adaboost with hierarchical structures is more preferable in anomaly detection of WSN.

**Table 3:** Comparison of Adaboost with and without hierarchical structures.

	Adaboost without hierarchical structures	Adaboost with hierarchical structures
*DR*(%)	92.35	92.40
*FP*(%)	8.50	1.66

#### Experiment 2

Evaluation of performance of mis-use detection of Sink nodes. This paper puts side by side different results of intrusion detection by the mean of 50 times experiments, based on CA-AFSA-BP, BP neural network, Support Vector Machine (SVN), Particle-Swarm-Optimization (PSO)-BP, demonstrated in Table 4. The performance of mis-use detection by CA-AFSA-BP excels other three algorithms. The detection of DoS attacks is as high as 98.03%, which means the mis-use detection by CA-AFSA-BP of DoS attacks is pretty effective. DR of U2R and R2L attacks are relatively low because of limited sample resources. Table 5 shows that mis-use detection algorithm the paper brings up has a high accuracy.

**Table 4:** Classification Results of 4 intrusion detection algorithms.

Type	BP	SVM	PSO-BP	CA-AFSA-BP
	*DR*(%)	*DR*(%)	*DR*(%)	*DR*(%)
Probe	78.91	75.65	73.91	76.30
DoS	82.50	96.28	97.87	98.03
U2R	6.45	9.68	12.90	19.35
R2L	29.45	26.71	20.54	30.14
Totle	78.03	87.41	87.96	89.55

**Table 5:** DR and FR of 4 intrusion detection algorithms.

	*FP*(%)	*Accuracy*(%)
BP	2.07	82.43
SVM	2.48	89.03
PSO-BP	1.86	89.60
CA-AFSA-BP	1.66	90.43

## Conclusions

At present, active defense system of intrusion detection has been a hot topic of research on WSN security. The paper introduces a two-level intrusion detection model of cluster-based WSN integrating anomaly detection inside clusters and mis-use detection of Sink nodes. Different detection is performed according to the availability of resources. Both algorithms demonstrate a relatively high detection rate and low false rate. There are still some deficiencies, such as update system of CA-AFSA-BP in mis-use detection is complicated and non self-learning, and the unknown attack, which is left out in anomaly detection, cannot be determined. Coming research work is dedicated to these questions. And we will also take node energy and information entropy [16–19] for WSN into account for the future research.
